# An equine iPSC-based phenotypic screening platform identifies pro- and anti-viral molecules against West Nile virus

**DOI:** 10.1186/s13567-024-01290-1

**Published:** 2024-03-16

**Authors:** Marielle Cochet, François Piumi, Kamila Gorna, Noémie Berry, Gaëlle Gonzalez, Anne Danckaert, Nathalie Aulner, Odile Blanchet, Stéphan Zientara, Francesc Xavier Donadeu, Hélène Munier-Lehmann, Jennifer Richardson, Alexandra Benchoua, Muriel Coulpier

**Affiliations:** 1 UMR VIROLOGIE, Laboratoire de Santé Animale, INRAE, Anses, Ecole Nationale Vétérinaire d’Alfort, 94700 Maisons-Alfort, France; 2 UTechS Photonics Bioimaging/C2RT, Institut Pasteur Paris, Université Paris Cité, 75015 Paris, France; 3 Centre de Ressources Biologiques, BB-0033-00038, CHU Angers, 49933 Angers, France; 4 Division of Translational Bioscience, The Roslin Institute and Royal (Dick) School of Veterinary Studies, University of Edinburgh, Easter Bush, Midlothian, EH25 9RG UK; 5 CNRS UMR3523, PF-CCB, Institut Pasteur, Université Paris Cité, 75015 Paris, France; 6 CECS, I-STEM, AFM, 91100 Evry, France

**Keywords:** Equine, brain, neural progenitors, Flavivirus, antiviral, statin, nucleoside analog

## Abstract

Outbreaks of West Nile virus (WNV) occur periodically, affecting both human and equine populations. There are no vaccines for humans, and those commercialised for horses do not have sufficient coverage. Specific antiviral treatments do not exist. Many drug discovery studies have been conducted, but since rodent or primate cell lines are normally used, results cannot always be transposed to horses. There is thus a need to develop relevant equine cellular models. Here, we used induced pluripotent stem cells to develop a new in vitro model of WNV-infected equine brain cells suitable for microplate assay, and assessed the cytotoxicity and antiviral activity of forty-one chemical compounds. We found that one nucleoside analog, 2′C-methylcytidine, blocked WNV infection in equine brain cells, whereas other compounds were either toxic or ineffective, despite some displaying anti-viral activity in human cell lines. We also revealed an unexpected proviral effect of statins in WNV-infected equine brain cells. Our results thus identify a potential lead for future drug development and underscore the importance of using a tissue- and species-relevant cellular model for assessing the activity of antiviral compounds.

**Supplementary Information:**

The online version contains supplementary material available at 10.1186/s13567-024-01290-1.

## Introduction

West Nile virus (WNV) is a neurotropic mosquito-borne arbovirus belonging to the *Flavivirus* genus of the *Flaviviridae* family that infects many species, including humans and equids. Although usually asymptomatic or causing mild flu-like symptoms, infection can sometimes lead to neuroinvasive diseases such as meningitis, encephalitis or poliomyelitis, which can be fatal for both humans and horses [1, 2]. In the last decades, WNV has expanded geographically and become endemic in many countries, causing an increase in the number of West Nile neuroinvasive disease (WNND) cases [3]. Although three vaccines are available for horses [4], their coverage is insufficient and outbreaks remain a regular occurrence [5, 6]. Specific antiviral drugs are not available and existing treatments are merely supportive.

Investigation of antiviral compounds has allowed identification of both direct-acting and host-directed antivirals, as outlined in recent reviews [7, 8]. Direct-acting antivirals mostly target the RNA-dependent RNA polymerase (RdRp) of the viral nonstructural protein 5 (NS5), acting through blockage of genome replication. Host-directed antivirals include virus entry, nucleoside biosynthesis and cyclophilin inhibitors, as well as compounds targeting proteins associated with the ER or lipid metabolism and anti-parkinsonism drugs, thus affecting the viral cycle at different stages. Host-directed antivirals would in principle have less tendency to induce selection of drug-resistant viruses and possibly a natural propensity to provide broad-spectrum activity, as host factors may be co-opted by different viruses. Nonetheless, broad-spectrum activity can also be exhibited by direct-acting antivirals, such as nucleoside analogs, as RdRps are highly conserved amongst RNA viruses.

The antiviral activity of direct-acting and host-directed antivirals has generally been evidenced using cell lines of rodent or primate origin, models that may be poorly predictive of therapeutic efficacy in horses. Equine dermal cells (ED) have been used to test antivirals against equine arteritis virus [9] and equine herpes virus [10], but are unsuitable for study of WNV as they are weakly permissive to WNV (personal observation). Equine brain cells would represent a more relevant model, as WNV disease results from brain infection. In humans, the study of viral brain infection has recently benefited from the development of brain cell models derived from induced pluripotent stem cells (iPSCs) [11, 12] or fetal neural progenitor cells (NPCs) [13–15]. The advent of iPSC technology has also provided opportunities for modelling equine diseases [16, 17], and indeed brain cells susceptible to WNV have recently been generated [17]. Such findings, however, have not yet been independently reproduced, and unlike in humans [12], the use of equine iPSC-derived cells for assessing antiviral potential has not been reported.

Our aim was to develop an equine brain cell-based model of WNV infection that could be used to identify therapeutic candidates with antiviral activity in horses. Of 41 chemical compounds tested, one displayed activity against WNV, whereas others were either inactive or exhibited, unexpectedly, pro-viral activity.

## Materials and methods

### Ethics statement

Human fetus was obtained after legal abortion with written informed consent from the patient. The procedure for the procurement and use of human fetal central nervous system tissue was approved and monitored by the “Comité Consultatif de Protection des Personnes dans la Recherche Biomédicale” of Henri Mondor Hospital, France. Authorization and declaration numbers at the Research Ministry are AC-2017-2993 (CHU Angers) and DC-2019-3771 (UMR Virologie). The rabbit immunization protocol (anti-WNV-E3 antibody) complied with EU legislation (authorization 12/04/11-6 accorded by the ANSES/ENVA/UPEC ethical committee).

### Cell culture

VERO (ATCC No. CRL-1586) and A549 (ATCC No. CCL-185) cells were cultured in Minimum Essential Medium (MEM, TFS, Fr) supplemented with 10% fetal bovine serum (FBS, TFS, Fr). Human neural progenitor cells (hNPCs) were prepared and cultured as described [18]. Equine iPSCs (eiPSCs) were obtained as described [16] and cultivated feeder-free using a matrix of truncated vitronectin (Vitro-N, Gibco, TFS, Fr) in a medium composed of StemMACS iPS-Brew (Miltenyi Biotech, Germany) supplemented with mouse LIF (1000 U/mL, Merck Millipore). Neural induction and collection, amplification and banking of eNPCs were achieved as described [19]. Equine NPCs were maintained on poly-ornithin/laminin coated dishes in N2B27-GF medium [DMEM-F12 with GlutaMAX:Neurobasal (1:1) plus N2, B27 without vitamin A and 0.55 mM 2-mercaptoethanol (TFS, Fr) supplemented with EGF, bFGF and BDNF (10–10–20 ng/mL, respectively, PeproTech)]. Neuronal differentiation of eNPCs (Passage 3 to 8) was induced by EGF and bFGF withdrawal 24 h after plating (125 000 cells/cm^2^ in 96-well plates, Greiner Bio-One). Medium was changed three times a week. All cells were maintained at 37 °C, 5% CO_2_. TFS, Thermo Fisher Scientific. Fr, France.

### Virus and infection

A stock of WNV_NY99_ strain (Genebank Accession No. KC407666.1) was generated in VERO cells, aliquoted and stored at −80 °C until use. Titer was estimated by plaque assay as described [20]. Cells were infected at the indicated MOI for 2 h at 37 °C before removal of the inoculum and replacement by fresh medium until collection of supernatants at indicated time. Virus titers in supernatants were estimated by endpoint dilution (TCID_50_) as described [21]. All procedures were performed under bio-safety level-3 conditions.

### Chemical compounds and screening assay

The forty-one chemical compounds (listed in Additional file 1 and described in [22, 23]) as well as fluvastatin, simvastatin and lovastatin (SML0038, S6196, 438185, Sigma) were reconstituted at 10 mM in dimethylsulfoxide (DMSO, Sigma) or water. All compounds were diluted to indicated concentrations in N2B27-GF medium containing 0.2% DMSO. Non-infected/WNV-infected eNPCs were used as negative/positive controls and maintained in N2B27-GF plus 0.2% DMSO. Compounds were added to the medium 2 h before WNV infection and maintained for the next 48 h, up until analysis. Supernatants were then collected and cells fixed in 4% paraformaldehyde (Electron Microscopy Science) for analyses.

### Immunofluorescence assays

Standard immunofluorescence was performed as described [14]. Primary antibodies against βIII-Tubulin (T8660-Sigma or Ab18207-Abcam), HuC/D (A-21271-TFS), GFAP (Z0304-DAKO), SOX2 (AB5603-Millipore), and the domain 3 of WNV envelope (WNV-E3, home-made) were used. Secondary antibodies were Alexa Fluor 488/546 anti-rabbit/anti-mouse (TFS, Fr). Nuclei were stained with 0.1 ng/mL 4,6-diamidino-2-phenylindole (DAPI, Sigma).

### Image acquisition and analysis

Two channel images were acquired in a fully automated and unbiased manner using the Opera Phenix™ High-Content Screening System (Revvity, Fr) and a 10× air objective (NA = 0.3). Thirty-five images per channel per condition were collected, transferred to the Columbus Conductor ™ Database and analyzed with Acapella software (Revvity, Fr), using a customized algorithm for cell segmentation. Approximately 32 000 cells were counted per well.

### Determination of the selectivity index

The experimental design described above for the screening assay, as well as in Figure 3A, was used to determine the half maximal inhibitory (IC50) and cytotoxicity (CC50) concentrations, along with the resulting selectivity index (SI = CC50/IC50) of compounds considered to be hits.

### RNA isolation and real time PCR

Procedures were as described [14]. Primers used were WNV-F, 5′CCTGTGTGAGCTGACAAACTTAGT-3′ and WNV-R, 5′GCGTTTTAGCATATTGACAGCC-3′.

### Statistical analysis

Statistical analyses were performed with GraphPad Prism V9.2.0. CC50, IC50 and SI were calculated with the R package “drc” (version 3.0.1) [24].

## Results

### Derivation of brain cells from equine induced pluripotent stem cells (eiPSCs) and their permissivity to WNV

**Figure 1:**
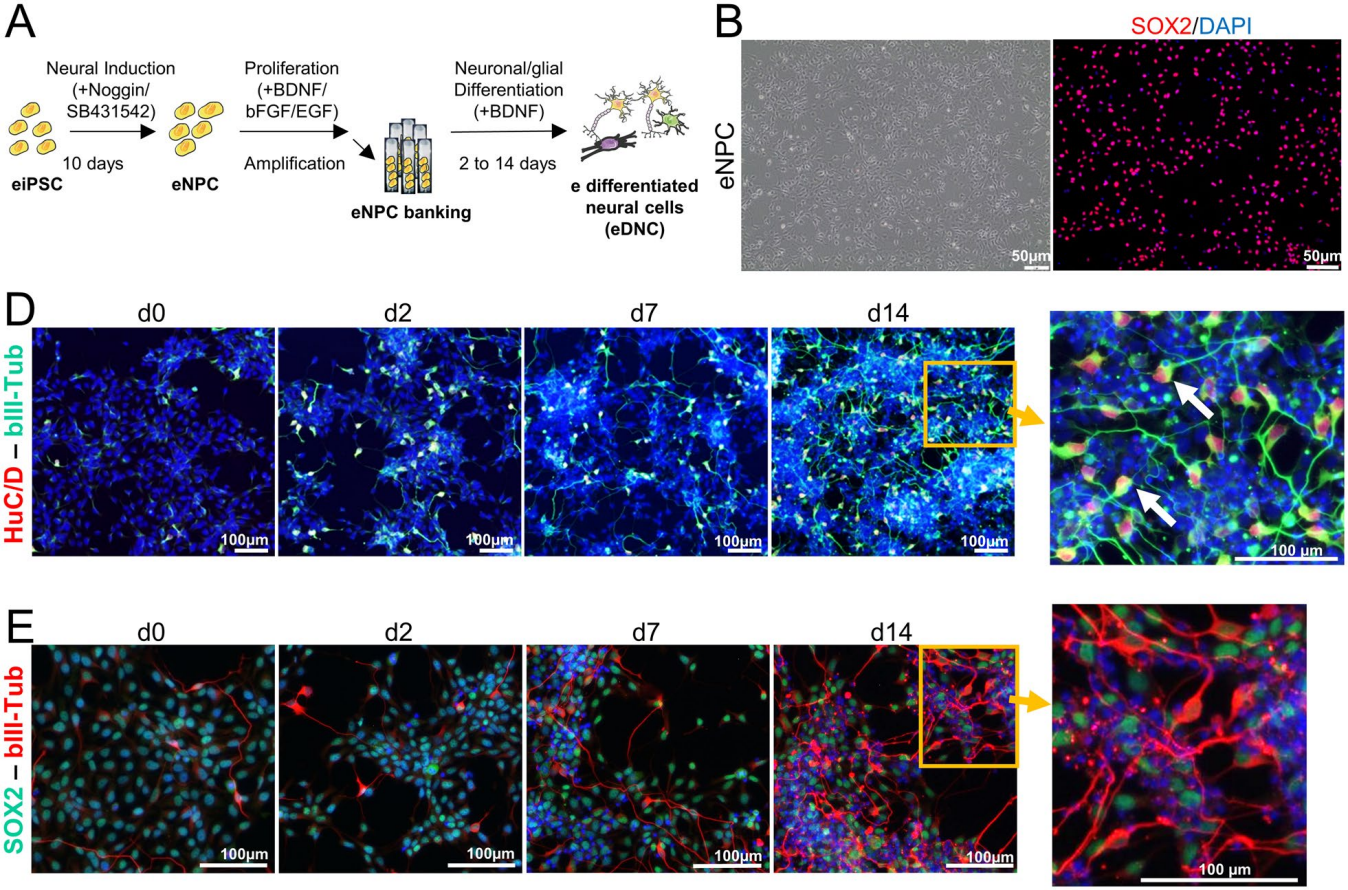
**Derivation of neural progenitors and neurons from equine iPSC (eiPSC).** **A** Schematic representation of the experimental procedure. **B** Bright field images (left) and immunofluorescence labeling of eNPCs with antibody directed against SOX2 (red, right). **C**, **D** Immunofluorescence labeling of equine brain cells from day 0 to day 14 of differentiation. **C** Antibodies directed against βIII-Tubulin (green) and HuC/D (red) reveal equine neurons. **D** Antibodies directed against SOX2 (green) and βIII-Tubulin (red) reveal eNPCs and eNe, respectively. Nuclei were stained with DAPI (blue). e: equine.

Equine iPSCs generated previously [16] were differentiated into the neural lineage as described (Figure 1A). Neural induction led to a stable population of proliferating eNPCs that homogeneously expressed the neural nuclear marker SOX-2 (Figure 1B). Further differentiation of eNPCs, achieved by bFGF and EGF withdrawal, allowed the progressive generation of equine neurons (eNe) over time, as revealed by immunostaining for two neuronal markers, βIII-Tubulin and huC/D (Figures 1D and E). Fourteen days after growth factors withdrawal, however, the differentiation process was incomplete, as progenitor cells (SOX-2 positive) were still numerous in the vicinity of young neurons (SOX-2/βIII-Tubulin-positive) (Figure 1E), and glial cells were not detected upon immunostaining for markers of astrocytes (GFAP) and oligodendrocytes (OLIG-2) (not shown). Also, extensive cell death, as revealed by the presence of numerous floating cells, occurred at that time, therefore precluding prolongation of the differentiation process.

**Figure 2:**
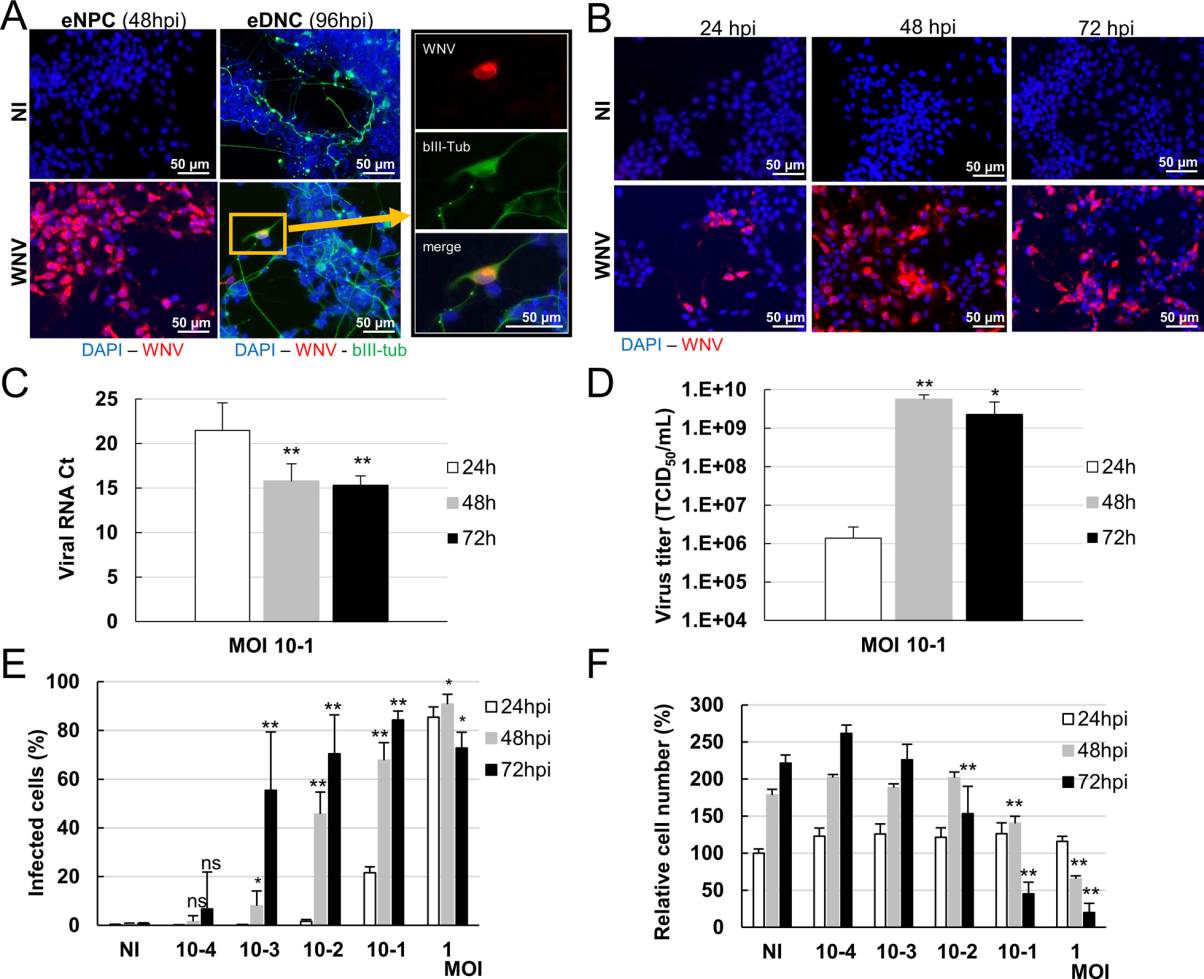
**Permissivity of equine neural progenitor cells and neurons to WNV.** **A**–**D** Equine NPCs and equine neural cells differentiated for 14 days from eNPCs (eDNCs) were infected with WNV_NY99_ at MOI 10^–1^. **A** Immunofluorescence labeling with antibodies against WNV-E3 (red) and βIII-Tubulin (green). **B** Immunofluorescence labeling with WNV-E3 antibody (red) shows virus spreading in eNPCs. Cells were stained with DAPI (blue). **C** Viral RNA from supernatant was analyzed by RT-qPCR. **D** Virus in supernatant was titrated by end-point dilution (TCID_50_). **E**, **F** Equine NPC were infected at MOI from 10^–4^ to 1 and enumerated automatically based on fluorescent staining using an OPERA instrument. Enumeration of **E** the percentage of WNV-infected eNPCs (immunostaining with WNV-E3 antibody) and **F** total eNPCs number (DAPI staining). Normalization was to non-infected eNPCs at 24 h (**F**). Results are pooled from 3 independent experiments performed in duplicate (**C**, **D**) or representative of 3 independent experiments performed in 6 replicates (**E**, **F**). Data are expressed as the mean ± SD. Comparison between cells infected for 48/72 h and cells infected for 24 h (**C**–**E**) and comparison between infected cells (at MOI from 10^–4^ to 1) and non-infected cells at the same time post-infection (**F**) were performed with a two-tailed unpaired Mann–Whitney test with *p*-values significant when *p < 0.05, **p < 0.01.

We thus next assessed the permissivity of equine brain cells at day 0 (eNPCs) and day 14 (equine differentiated neural cells-eDNCs) of differentiation. Equine NPCs and eDNCs were infected with WNV_NY99_ at MOI 10^–1^ for 48 h and 96 h, respectively. Immunostaining with anti-WNV-E3 antibody revealed clusters of massively infected eNPCs (Figure 2A, left) whereas infection of eNe, albeit detected, was rare (Figure 2A, right). We therefore decided to use eNPCs for antiviral screening and aimed at further characterizing their infection while defining optimal conditions for screening. Kinetic studies performed between 24 and 72 h with WNV_NY99_ at MOI 10^–1^ demonstrated virus replication and spread in eNPCs, as shown by immunofluorescence labeling with WNV-E3 antibody (Figure 2B) and quantification of viral RNA and viral titers in supernatants by RT-qPCR (Figure 2C) and end-point dilution (Figure 2D), respectively. Dose response studies performed with WNV_NY99_ at MOI ranging from 10^–4^ to 1, associated to automated quantification of the percentage of infected cells and total cell number, showed dose-dependent changes in viral infection (Figure 2E) and survival (Figure 2F), with deep alteration of eNPCs growth and survival at the highest MOI. Of note, at MOI 10^–1^, increase in viral infection from 24 to 72 hpi was similarly detected by RT-qPCR, titration and image analysis (Figures 2C–E), establishing that image-based analysis is a suitable method to quantify viral infection in eNPCs. Based on these results, we established the screening conditions (MOI 10^–2^ for 48 h) such as there is a substantial percentage of infected cells (approximately 45%) with no impact on cell growth or survival.

### Phenotypic screen using eNPCs identifies compounds with antiviral and proviral properties

**Figure 3:**
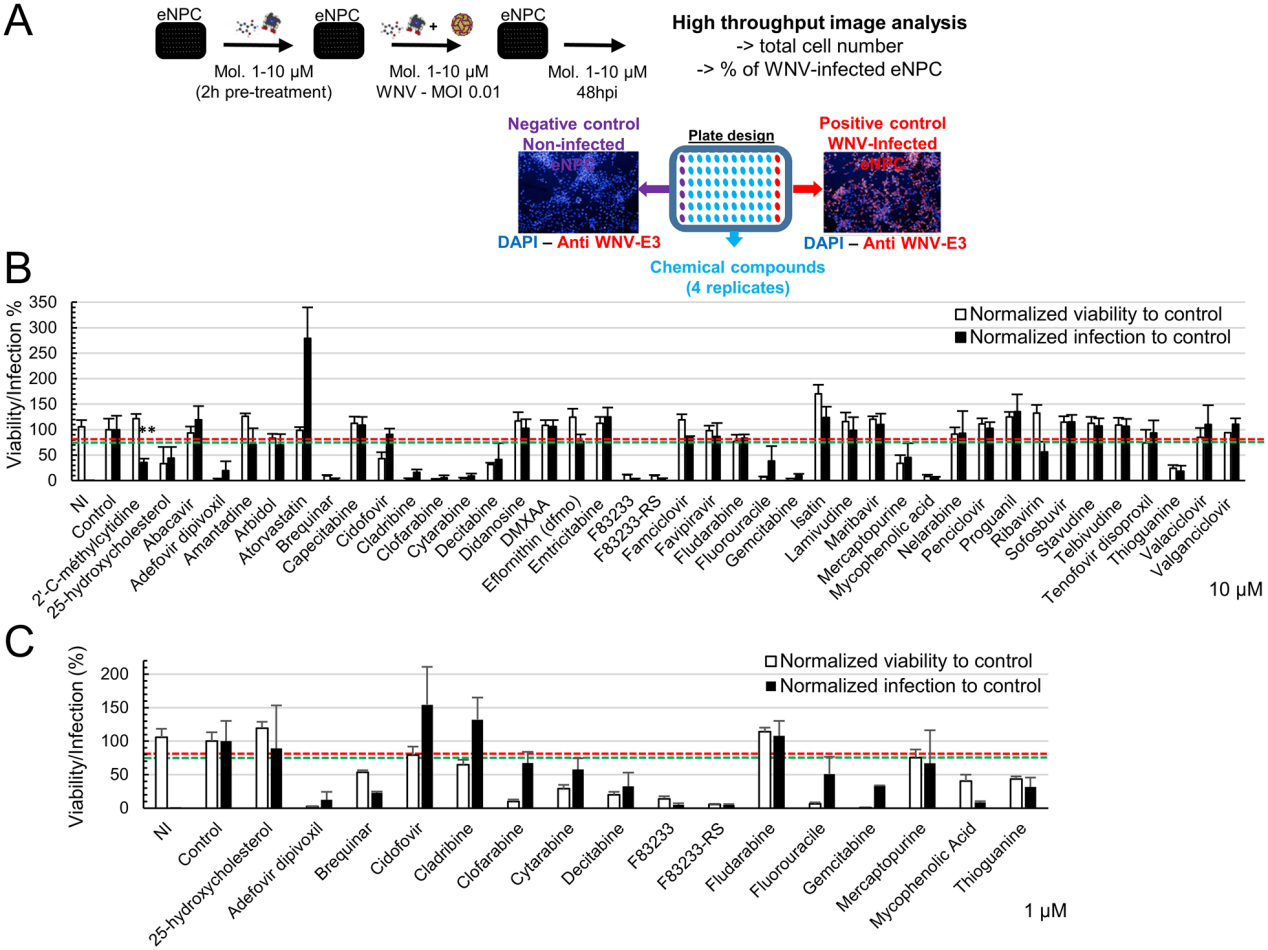
**Screen of 41 compounds for their antiviral activity against WNV in eNPCs.** **A** Schematic representation of the phenotypic screen. Mol., Molecule (**B**) Screen of 41 compounds at 10 µM. **C** Screen of 16 compounds at 1 µM. A hit was arbitrarily defined as reducing infection by 25% (below the green dashed line) and showing less than 20% toxicity (above the red dashed line). Results are representative of 2 independent screens (at each dose) performed in quadruplicate. Total number of cells and percentage of infected cells were normalized to control (non-treated WNV-infected eNPCs). Data are expressed as the mean ± SD. Comparison between WNV-infected treated and non-treated cells was performed with a two-tailed unpaired Mann–Whitney test with p-values significant when *p < 0.05, **p < 0.01.

**Figure 4:**
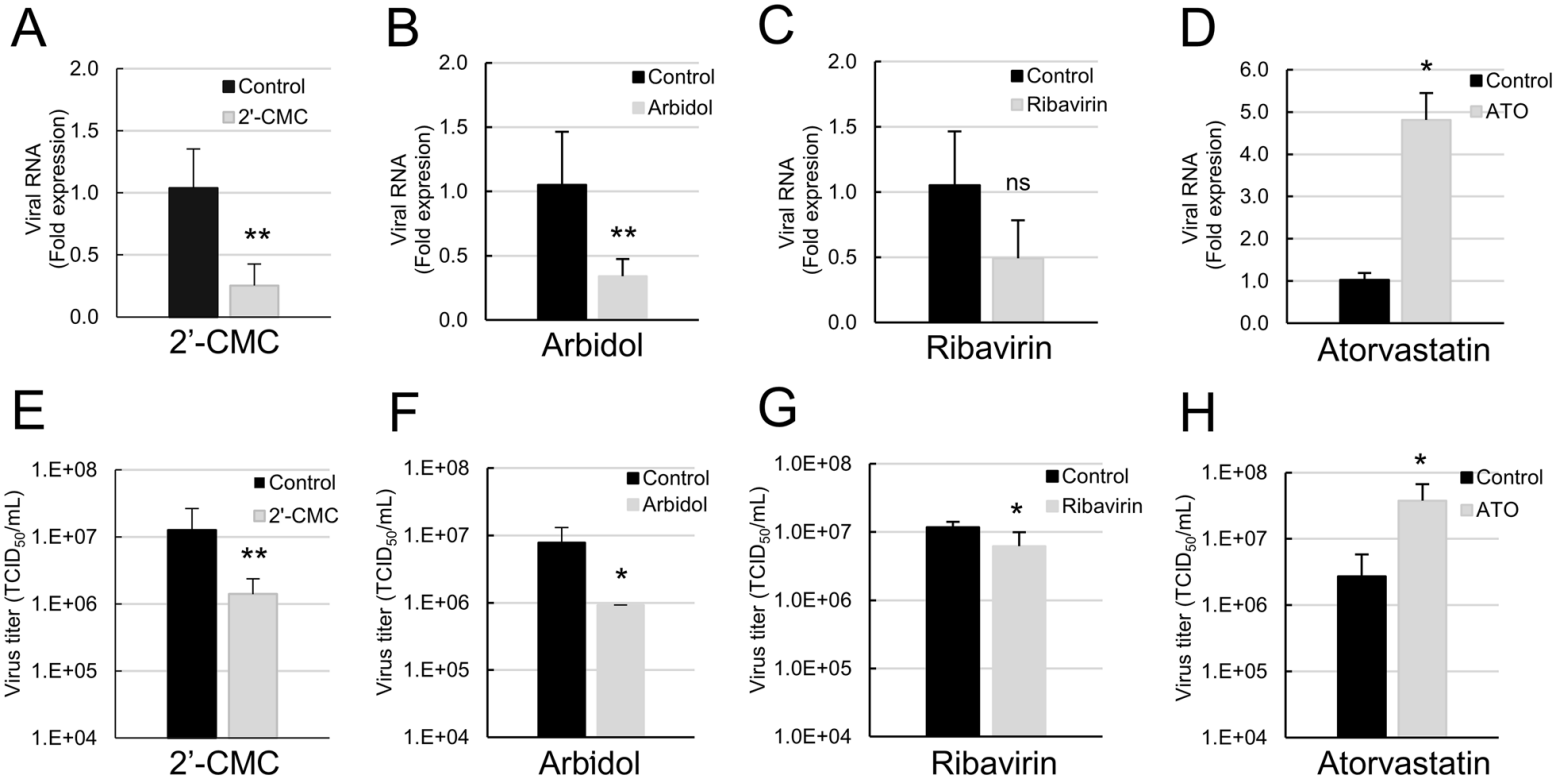
**Anti- and pro-viral effects of 2′CMC, arbidol, ribavirin, and atorvastatin in WNV-infected eNPCs.** Supernatants of WNV-infected eNPCs, non-treated or treated with compounds at 10 µM, were collected 48 hpi and analyzed for **A**–**D** viral RNA expression (RT-qPCR) and **D**–**F** virus titer (TCID_50_/mL). Results are pooled from 2 independent experiments performed in triplicate. They are expressed as the mean ± SD. Comparison between WNV-infected treated and non-treated eNPCs was performed with a two-tailed unpaired Mann Whitney test with *p*-values significant when *p < 0.05, **p < 0.01.

WNV_NY99_-infected eNPCs were used to screen a library of 41 chemical compounds selected for their antiviral activity against human and equine viruses (Additional file 1), as schematically represented (Figure 3A). Toxicity and antiviral effect were determined by automated quantification of total cells labeled with DAPI and infected cells immuno-labelled with WNV-E3 antibody, respectively. A hit was arbitrarily defined as a compound inducing a reduction of at least 25% of infected cells and less than 20% cell loss. Of all compounds tested at 10 µM (Figure 3B), 39% (16/41) were toxic, suggesting that eNPCs were particularly sensitive to drugs. Among the non-toxic molecules (25/41), 21 (amantadine, capecitabine, DMXAA, eflornithin, favipiravir, herpes virus and reverse transcriptase inhibitors, isatin, maribavir, nelarabine and sofosbuvir) had no antiviral activity against WNV. Three compounds, 2′C-methylcytidine (2′-CMC), arbidol and ribavirine, reduced the percentage of infected cells to 35.3 ± 7.5%, 69.7 ± 21.3% and 56 ± 20.1%, respectively, and were thus considered to be hits, and surprisingly, one of the compounds, atorvastatin, induced an increase in the percentage of infected cells (279.2 ± 60.6%), revealing a pro-viral effect. For compounds exerting toxicity at 10 µM, an additional screen was performed at 1 µM (Figure 3C). Although cytotoxicity was generally reduced, cellular loss remained above 20% for all but two compounds, fludarabine and 25-hydroxycholesterol, which nonetheless showed no antiviral activity. Of note, mycophenolic acid and brequinar displayed strong antiviral activity, albeit inducing 50% or greater cell loss (Figure 3C). These results are summarised (Table 1). In order to verify the effect of the 4 compounds identified as modulators of WNV infection, we next quantified viral RNA and infectious viral particles in supernatants of eNPCs treated or not with 10 µM of 2′-CMC, arbidol, ribavirin or atorvastatin (Figure 4). 2′-CMC and arbidol induced a decrease in both viral RNA (Figures 4A, B) and viral titers (Figures 4E, F), confirming their antiviral impact. This was not the case of ribavirin, for which a decrease in viral RNA was not detected (Figure 4C), despite a decrease in virus titer (Figure 4G). The proviral effect of atorvastatin was confirmed when quantifying both viral RNA and infectious viral particles (Figures 4D, H). Thus, our newly developed screen based on image analyses allowed efficient identification of molecules that inhibit or promote WNV replication in equine brain cells, as well as simultaneous assessment of their toxicity.

**Table 1:** **Antiviral and toxicity activities of compounds on WNV-infected eNPCs**

Compounds	Type	Viral replication	Cytotoxicity (10 µM)
2′-C-methylcytidine	DAA-NA	AV	
25-Hydroxycholesterol	HDA		Tx
Abacavir	DAA-RTI	NoA	
Adefovir dipivoxil	DAA-HVI		Tx
Amantadine	HDA	NoA	
Arbidol	HDA	AV	
Atorvastatin	HDA	PV	
Brequinar	HDA		Tx
Capecitabine	N/A	NoA	
Cidofovir	DAA-HVI		Tx
Cladribine	N/A		Tx
Clofarabine	N/A		Tx
Cytarabine	N/A		Tx
Decitabine	DAA-NA		Tx
Didanosine	DAA-RTI	NoA	
DMXAA	HDA	NoA	
Eflornithin (dfmo)	HDA	NoA	
Emtricitabine	DAA-RTI	NoA	
F83233	HDA		Tx
F83233RS	HDA		Tx
Famciclovir	DAA-HVI	NoA	
Favipiravir	DAA-NA	NoA	
Fludarabine	DAA-NA		Tx
Fluorouracile	DAA-NA		Tx
Gemcitabine	DAA-NA		Tx
Isatin	HDA	NoA	
Lamivudine	DAA-RTI	NoA	
Maribavir	DAA	NoA	
Mercaptopurine	DAA-NA		Tx
Mycophenolic acid	HDA		Tx
Nelarabine	N/A	NoA	
Penciclovir	DAA-HVI	NoA	
Proguanil	HDA	NoA	
Ribavirin	DAA-NA, HDA	AV	
Sofosbuvir	DAA-NA	NoA	
Stavudine	DAA-RTI	NoA	
Telbivudine	DAA-RTI	NoA	
Tenofovir disoproxil	DAA-RTI	NoA	
Thioguanine	DAA-NA		Tx
Valaciclovir	DAA-HVI	NoA	
Valganciclovir	DAA-HVI	NoA	

DAA: direct-antiviral activity, HDA: host-directed antiviral, NA: nucleoside analog, RTI: reverse transcriptase inhibitor, HVI: herpes virus inhibitor, Tx: toxic, AV: antiviral activity, NoA: no activity, PV: pro-viral activity.

### Selectivity index for 2′-CMC, arbidol and ribavirin

**Figure 5:**
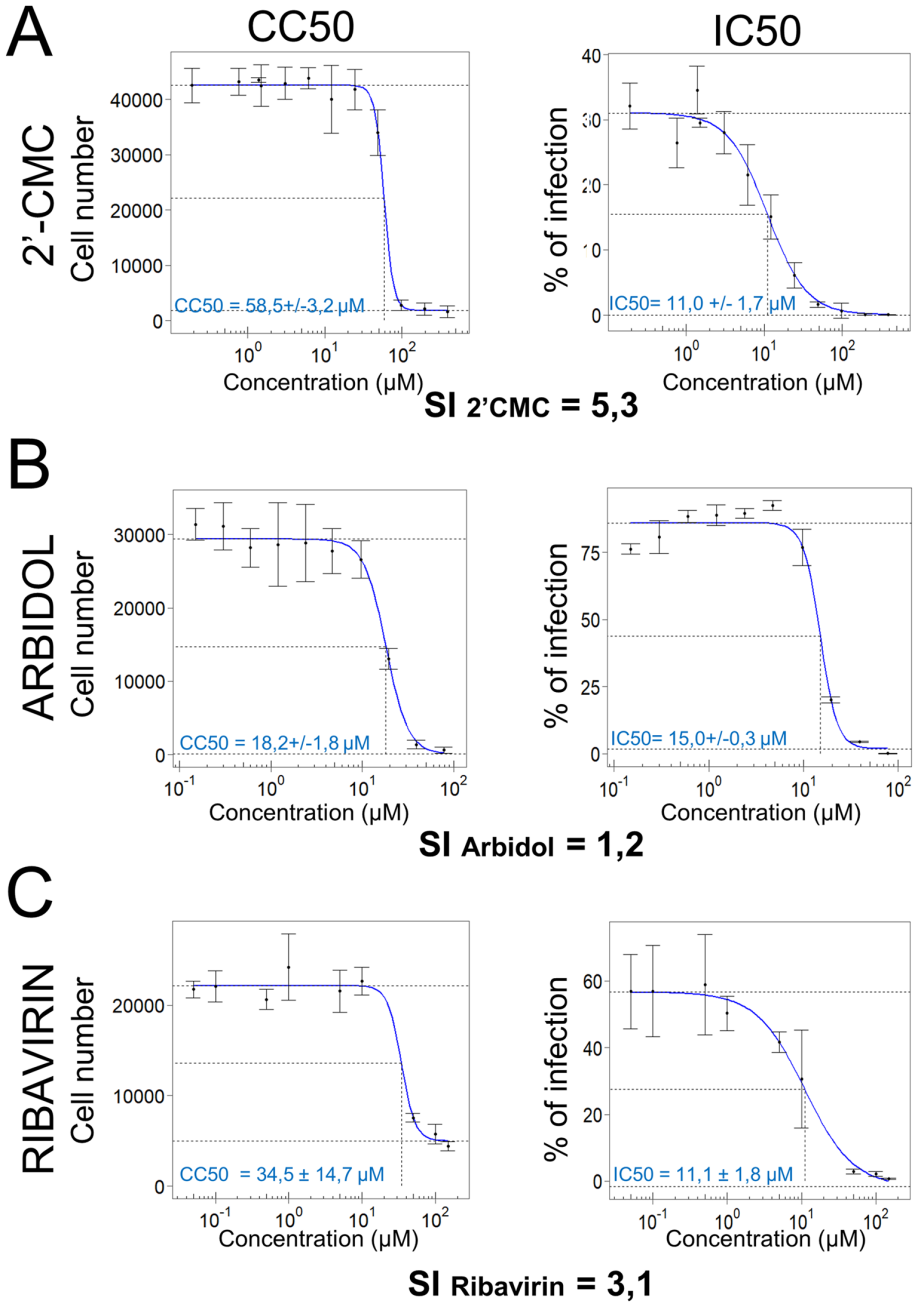
**Selectivity index of 2′CMC, arbidol and ribavirin.** WNV-infected eNPCs were treated with increasing concentration of selected compounds (from 0.2 to 390 µM for 2′-C-methylcytidine, from 0.15 to 78 µM for arbidol and from 0.05 to 150 µM for ribavirin) and analysed at 48 hpi. Total and infected cells were enumerated automatically based on DAPI staining and immunostaining with an anti-WNV-E3 antibody. **A** 2′CMC, **B** arbidol and **C** ribavirin. Results are representative of 3 independent experiments performed in triplicate. They are expressed as the mean ± SD. CC: cytotoxicity concentration, IC: inhibitory concentration, SI: selectivity index.

Using the experimental design described (Figure 3A), dose responses were evaluated, and IC50, CC50 and SI (CC50/IC50) determined for 2′-CMC, arbidol and ribavirin. As shown (Figure 5), each drug was effective in the 10 micromolar range, with IC50 being 11 ± 1.7 µM, 15 ± 0.3 µM and 11.1 ± 1.8 µM for 2′-CMC (Figure 5A), arbidol (Figure 5B), and ribavirin (Figure 5C), respectively. 2′-CMC presented the highest SI (5.3) and arbidol the lowest (1.2), revealing for the latter a toxicity in the same range of concentrations as antiviral activity.

### Atorvastatin, simvastatin, lovastatin and fluvastatin have a pro-viral effect on WNV-infected eNPCs

**Figure 6:**
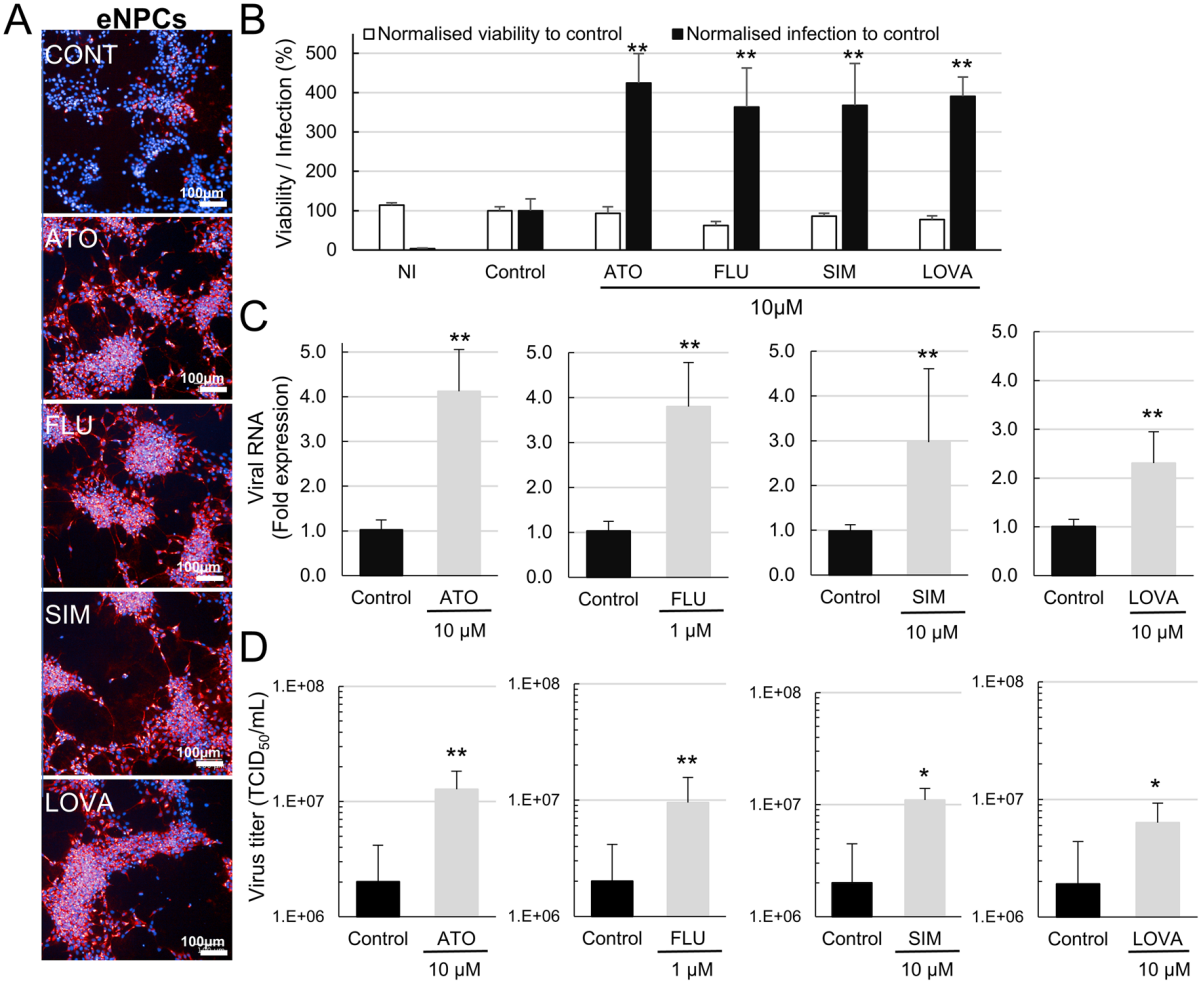
**Statins have a proviral role in WNV-infected eNPCs.** WNV-infected eNPCs (MOI 5 × 10^–3^) were treated with 1 or 10 µM atorvastatin, fluvastatin, simvastatin or lovastatin. **A** Photomicrographs of WNV-infected eNPCs treated or not with statins. Cells were immunostained with anti-WNV-E3 antibody (red). Nuclei were stained with DAPI (blue). **B** Automated enumeration of total cell number based on DAPI staining and percentage of infected cells based on immunostaining with anti-WNV-E3 antibody. Normalisation to control (non-treated WNV-infected eNPCs). Quantification of **C** viral RNA and **D** virus titers in supernatant. Results are representative of 3 independent experiments performed in quadruplicate (**A**, **B**) and are pooled from 2 independent experiments performed in triplicate (**C**, **D**). They are expressed as the mean ± SD. Comparison between treated and non-treated WNV-infected eNPCs was performed with a two-tailed unpaired Mann Whitney test with *p*-values significant when *p < 0.05, **p < 0.01. ATO: atorvastatin, FLU: fluvastatin, SIM: simvastatin, LOVA: lovastatin.

The proviral effect of atorvastatin in WNV-infected eNPCs raised the question of whether other statins may act similarly. We thus infected eNPCs with WNV_NY99_ at MOI 5 × 10^–3^ for 48 hpi (approximately 25% of cells were infected) and treated them as previously described with 3 additional statins: fluvastatin, simvastatin and lovastatin, all at 10 µM. All statins induced an increase in the percentage of infected eNPCs compared with non-treated cells (Figures 6A, B). Confirmation of their proviral role was obtained for all by quantification of viral RNA (Figure 6C) and infectious viral particles (Figure 6D) in the supernatants. In these latter experiments, fluvastatin was used at 1 µM (Figures 6C, D), in order to avoid any bias due to toxicity when used at 10 µM (Figure 6B).

### Statins have no effect or an anti-viral effect on WNV-infected VERO, A549 and human NPCs

**Figure 7:**
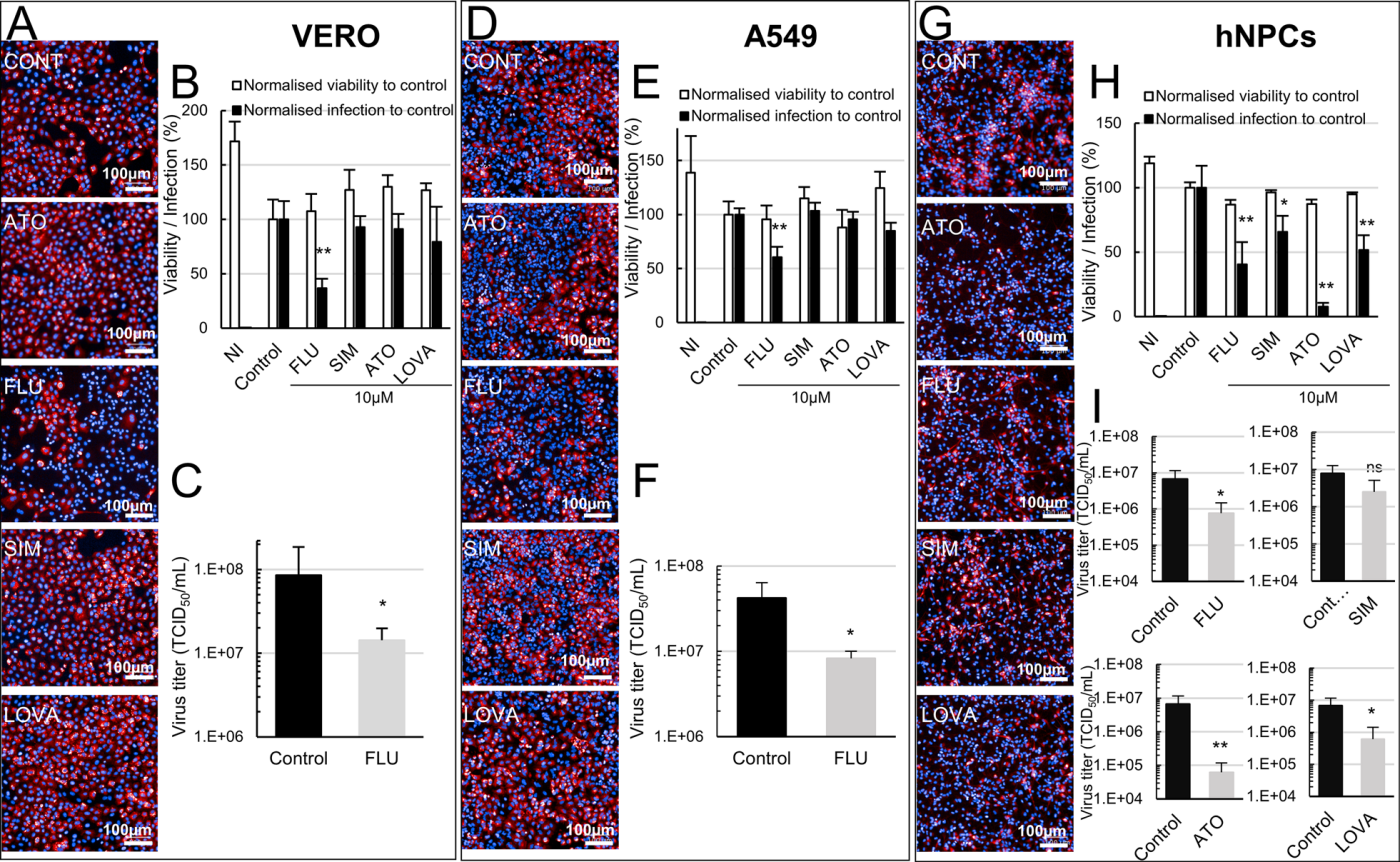
**Statins have no role or an anti-viral role in WNV-infected VERO, A549 cells and hNPCs.** WNV-infected cells (MOI 10^–2^) were treated with 10 µM fluvastatin, simvastatin, atorvastatin or lovastatin as shown in Figure 3A. Analysis was performed at 72 hpi (VERO) or 48 hpi (A549, hNPC). **A**, **D**, **G** Photomicrographs of WNV-infected VERO (**A**), A549 (**D**) and hNPCs (**G**) treated or not with statins. Cells were immunostained with anti-WNV-E3 antibody (red). Nuclei were stained with DAPI (blue). **B**, **E**, **H** Automated enumeration of total cell number based on DAPI staining and percentage of infected cells based on immunostaining with an anti-WNV-E3 antibody. Quantification of virus titers (**C**, **F**, **I**) in supernatants of VERO (**C**), A549 (**F**) and hNPCs (**I**). Results are representative of 3 independent experiments performed in quadruplicate (**B**, **E**, **H**) and are pooled from 2 independent experiments performed at least in duplicate (**C**, **F**, **I**). They are expressed as the mean ± SD. Comparison between treated and non-treated WNV-infected eNPCs was performed with a two-tailed unpaired Mann Whitney test with *p*-values significant when *p < 0.05, **p < 0.01. ns, non-significant (*p* > 0.05).

Given that statins have been described to have broad spectrum antiviral activity [25], the observation of a pro-viral effect in WNV-infected eNPCs was striking. To clarify their role, we further assessed statin activity in 2 cell lines (VERO and A549) and in NPCs of human origin (hNPCs) using an experimental design similar to that described in Figure 3A. Of all statins tested (at 10 µM), an effect in VERO (Figures 7A–C) and A549 (Figures 7D–F) cells was observed only for fluvastatin, which induced a decrease of 61% and 37% in the percentage of infected cells, respectively (Figures 7A and E). This was confirmed by quantification of virus titer in supernatant (Figures 7C and F). In hNPCs, all four statins exerted an antiviral effect, with atorvastatin having the strongest impact, as determined by fluorescent microscopy and quantification of infected cells (Figures 7G and H). For all statins except simvastatin, these findings were confirmed by quantification of virus titers in supernatants (Figure 7I). Thus, our results revealed differential effects of statins depending on cellular type and species, with a proviral effect that is specific to NPCs of equine origin.

## Discussion

WNV is a global health threat for both human and equine populations against which no antiviral drugs are currently available. The ability to derive brain cells from iPSCs provides an exciting new platform for in vitro modelling of equine disease and therapeutic screening [16, 17]. Here, we developed a novel microplate assay based on WNV-infected eNPCs and screened a chemical library of 41 compounds for their therapeutic potential. Compounds with antiviral, and unexpectedly, proviral activity, were found.

Upon infecting equine iPSC-derived brain cells, we found that eNe were rarely infected. As our protocol led to the generation of cortical neurons [19] which, in contrast to neurons from other brain areas such as thalamus, hindbrain, pons, medulla and ventral horn of the spinal cord, are weakly infected in equids [20], it is highly probable that the rare infection of eNe in our cultures reproduces the in vivo situation. The differential sensitivity of neuronal subtypes to WNV was also observed in an experimental murine model [21, 26]. Our results, however, are in striking contrast with those from Fortuna et al. [17], who observed a high infection rate in iPSC-derived equine cortical neurons. This discrepancy may be related to the use of different virus strains (WNV_NSW2011_ vs WNV_NY99_), with WNV_NSW2011_ being capable of infecting cortical areas in equine brains (although, to our knowledge, this was has not been reported). The high level of infection that we observed in eNPCs was also in contrast with the non-permissivity of eNPCs in Fortuna’s study. Our results are nevertheless in line with those observed in human NPCs, which are known to be highly permissive to a wide range of viruses [13, 27, 28].﻿

We demonstrated for the first time the feasibility of using WNV-infected equine brain cells to screen for antiviral activity and cytotoxicity in a microplate assay. Among the 41 chemical compounds evaluated in our pilot study, 2′-CMC, a nucleoside analog known to inhibit the viral polymerase of several flaviviruses [29–31], had the most robust activity. As no antiviral activity against WNV had been previously described for this compound, our results broaden its spectrum of action within the *Flavivirus* genus and across species, and identify a lead candidate, which calls for assessment of the antiviral activity of additional 2′C-methylated nucleoside derivatives. Ribavirin is another nucleotide analog identified in our screen. It is known to have a moderate effect on flaviviruses, and conflicting data have been reported in human neural progenitors [32, 33].﻿﻿ Our results support a similar moderate antiviral effect in WNV-infected eNPCs. Of note, two other nucleoside analogs, favipiravir and sofosbuvir, which also inhibit the viral polymerase and block WNV in human cell lines [34, 35], were inactive in eNPCs. It is possible that favipiravir, which generally inhibits viral replication in the 100 µM range, was inactive due to the lower dose used in our study (10 µM). Such, however, should not be the case for sofosbuvir, as its activity was observed in the micromolar range in 3 human cell lines [35]. Its inactivity in eNPCs may thus rather be attributed to insufficient uptake or conversion of the compound into its tri-phosphate active form, or alternatively, to its rapid elimination following extensive deamination or demethylation. This differential impact of a viral polymerase inhibitor on WNV-infected eNPCs and human cell lines underscores the importance of assessing the antiviral activity of compounds, including those acting directly against the virus, on relevant cell types and species.

The finding that statins had a proviral effect in WNV-infected eNPCs was unexpected, as statins had so far been shown rather to block the replication of viruses, including WNV and other flaviviruses, in multiple cellular types (reviewed in [25]). Our result did not appear to be related to neural progenitor cells in general, as statins can inhibit replication of both WNV (our study) and Japanese encephalitis virus [36] in human neural progenitors. Rather, the proviral effect might be specific to equids, possibly in relation to differential interaction with the human and equine hydroxymethylglutaryl-CoA reductase (HMG-CoA R) enzyme, the described target of statins, whose inhibition leads to a decrease in cellular cholesterol. Similar host species-specific effects in the inhibition of viral replication have already been documented for other small molecule compounds [37]. At present, the molecular mechanisms that underlie the proviral effect of statins in equine brain cells remain to be unravelled, but our unexpected observation further underscores the importance of using appropriate cell type and species-relevant models for assessing the role of antiviral molecules, and notably to avoid pointless pre-clinical and clinical assays.

Viral encephalitis continues to pose a significant threat for equids [38]. The discovery of effective antiviral compounds will most certainly rely on the development of relevant in vitro models. Our study demonstrates the feasibility of using equine iPSC-derived brain cells for studying viral infection and identifying suitable therapeutic compounds, providing a valuable discovery platform for the veterinary community that can be applied to diverse neurotropic equine viruses. A certain limitation, however, exists in the use of eNPCs rather than eNe, the latter being the main targets of WNV in equine brains [39]. Nonetheless, our results pave the way for the development of more sophisticated in vitro 2D and 3D models for which protocols are available in humans such as iPSC-derived motoneurons [40] and cerebral organoids [41], which more widely account for different cell populations of the brain.

### Supplementary Information

**Additional file 1.** **List of 41 selected compounds tested for their antiviral activity in WNV-infected eNPCs.**

## Data Availability

All data generated or analysed in this study are included in this article, except for those pertaining to immunostaining of GFAP and OLIG2, which are available upon request.
