# Supplementary material for: An equine iPSC-based phenotypic screening platform identifies pro- and anti-viral molecules against West Nile virus
Source: Vet Res. 2024 Mar 16;55:32. doi: 10.1186/s13567-024-01290-1 (PMC10943879; doi:10.1186/s13567-024-01290-1)
Supplement: Supplementary file 1 — Additional file 1. List of 41 selected compounds tested for their antiviral activity in WNV-infected eNPCs. [file 13567_2024_1290_MOESM1_ESM.docx]

**Additional file 1. List of 41 selected compounds tested for their antiviral activity in WNV-infected eNPCs.**

| **Compounds** | **Suppliers** | **Catalogue No.** |
| --- | --- | --- |
| 2’-C-methylcytidine | Ark Pharm, Inc | AK-88474 |
| 25-hydroxycholesterol | Cayman Europe | 11097 |
| Abacavir | TargetMol | T6367 |
| Adefovir dipivoxil | TargetMol | T1675 |
| Amantadine | Sigma | A1260 |
| Arbidol | TargetMol | T0104 |
| Atorvastatin | Sigma | PZ0001 |
| Brequinar | Adooq Bioscience | A12442 |
| Capecitabine | TargetMol | T1408 |
| Cidofovir | TargetMol | T6244 |
| Cladribine | TargetMol | T2558 |
| Clofarabine | TargetMol | T0297 |
| Cytarabine | TargetMol | T1272 |
| Decitabine | TargetMol | T1508 |
| Didanosine | TargetMol | T2578 |
| DMXAA | TargetMol | T6273 |
| Eflornithin (dfmo) | Sigma | D193 |
| Emtricitabine | TargetMol | T6214 |
| F83233 | In-house | (Ref 21) |
| F83233RS | In-house | (Ref 21) |
| Famciclovir | TargetMol | T1646 |
| Favipiravir | TargetMol | T6833 |
| Fludarabine | TargetMol | T1038 |
| Fluorouracile | AK Scientific, Inc. | AK-67526 |
| Gemcitabine | TargetMol | T0251 |
| Isatin | Sigma | 114618 |
| Lamivudine | TargetMol | T0682 |
| Maribavir | TargetMol | T2162 |
| Mercaptopurine | TargetMol | T0010 |
| Mycophenolic acid | Sigma | M5255 |
| Nelarabine | AK Scientific, Inc. | R077 |
| Penciclovir | TargetMol | T1643 |
| Proguanil | Bionet/keyorganic Ltd | HS-0102 |
| Ribavirin | MedChemExpress | HY-B0434 |
| Sofosbuvir | TargetMol | T6676 |
| Stavudine | TargetMol | T1404 |
| Telbivudine | TargetMol | T1513 |
| Tenofovir disoproxil | TargetMol | T2409 |
| Thioguanine | TargetMol | T3089 |
| Valaciclovir | TargetMol | T1087 |
| Valganciclovir | TargetMol | T1533 |
